# A longitudinal study of the correlation between coping style and post-traumatic growth in children and adolescents with a primary diagnosis of leukemia

**DOI:** 10.3389/fpsyt.2025.1664356

**Published:** 2025-10-16

**Authors:** Shouju Zhao, Xujia Xiao, Cheng Yang, Min Xu, Xianqiong Feng

**Affiliations:** ^1^ West China School of Nursing, Sichuan University/Department of Pediatric Intensive Care Unit Nursing, West China Second University Hospital, Sichuan University, Chengdu, China; ^2^ Key Laboratory of Birth Defects and Related Diseases of Women and Children (Sichuan University), Ministry of Education, Chengdu, China; ^3^ The Affiliated Hospital of Chengdu University, Chengdu, China; ^4^ Department of Nursing, West China Hospital, Sichuan University/West China School of Nursing, Sichuan University, Chengdu, Sichuan, China

**Keywords:** children, leukemia, post-traumatic growth, coping style, longitudinal study

## Abstract

**Objective:**

To investigate the types of coping styles and changes in post-traumatic growth (PTG) among children and adolescents with leukemia at different treatment stages and to analyze the correlation between these factors.

**Methods:**

A convenience sampling method was used. A general information questionnaire, Revised Post-traumatic Growth Inventory for Children, and Coping with Disease scale were administered to 60 children diagnosed with leukemia before induction of remission (T1), after induction of remission but before consolidation therapy (T2), and after consolidation therapy but before maintenance therapy (T3).

**Results:**

The level of PTG in children with leukemia at the initial diagnosis was moderate, showed an upward trend, and then stabilized. Statistically significant differences were observed in the total PTG and new possibilities dimension scores at the three time points (*P* < 0.05). The most common coping style was “Acceptance,” followed by “Cognitive–Palliative,” while “Avoidance” was the least frequently used. The Acceptance dimension scores showed statistically significant differences at all three time points (*P* < 0.05). At all three time points, the Acceptance, Cognitive–Palliative, and Avoidance dimensions were positively correlated with the total PTG score (*P* < 0.05). At T2 and T3, the Avoidance dimension score was positively correlated with the total PTG score and all dimensions except Appreciation of Life (*P* < 0.05). At T3, the Emotional Reaction dimension was negatively correlated with total PTG score, relating to the Others and Personal Strength dimensions (*P* < 0.05).

**Conclusion:**

The growth levels of children and adolescents with newly diagnosed leukemia improved in the early stages of treatment following trauma; however, this improvement gradually leveled off as the disease progressed. A positive coping style can positively influence PTG, whereas a negative coping style is detrimental to psychological development. Therefore, healthcare providers should comprehensively, scientifically, and dynamically assess the coping styles and behavioral manifestations of patients during treatment, focusing on fostering a positive coping style that promotes psychological growth and enhances mental health.

## 1 Introduction

Children’s cancer poses a severe threat to their health, with data released by the World Health Organization showing that nearly 40,000 children aged 0–19 develop cancer each year (1). According to the “National Children’s Tumor Surveillance Annual Report 2020,” leukemia has the highest incidence rate among various tumors, at 57.21% (2). By 2021, over 39,000 children in China were registered as having leukemia (3). Recently, with significant improvements in leukemia treatment efficacy, the 5-year survival rate of these children has exceeded 80% (4). Currently, chemotherapy is an effective treatment for leukemia; however, prolonged chemotherapy and the disease itself can impose a severe symptom burden on patients (5, 6), affecting their quality of life and mental health. Research has found that cancer and its treatments bring negative experiences and lead to positive changes under certain conditions (7–11). These positive changes are referred to as post-traumatic growth (PTG). PTG, a concept developed under the guidance of positive psychology, describes the transformative process by which individuals rebuild their psychological world after experiencing severe challenges to themselves or their inner world, thereby understanding and finding meaning in the traumatic experience (12). Literature indicates that PTG can help adolescents cope positively with traumatic events by guiding them to reassess their abilities and possibilities, thereby promoting mental health (13). However, most PTG studies on children with leukemia are based on the current situation (7, 13, 14), and it is unclear what dynamics and characteristics of PTG change with treatment time.

A review of the literature revealed that the generation and development of PTG is influenced by numerous factors, with an individual’s coping process being one of the most critical determinants of post-traumatic psychological and physical responses (15). Coping style refers to the methods, means, or tactics individuals use to address internal and external environmental demands along with related emotional distress (16). Different coping styles can lead to different outcomes when individuals experience stress or stressful events. Research has found that coping does not directly alter patients’ physical symptoms as a key mechanism influencing the outcome of stress responses, but significantly improves their mental state (17). Currently, coping styles are primarily categorized as positive or negative. Positive coping styles include facing confrontation, positive cognitive coping, and problem-oriented coping. Negative coping styles include submission, defense, and avoidance (18, 19). Among these, positive cognition and problem-solving in response to traumatic events are crucial factors for successful adaptation and prerequisites for the development of PTG (20). A survey conducted by domestic scholars such as Liu Q et al. (7) found that a positive coping style is an important protective factor against PTG in children and adolescents who are cancer survivors, whereas a negative coping style is a risk factor (18, 21). Given the prolonged course and extended treatment duration of pediatric leukemia, it is unclear what coping styles children adopt during this period and how these coping styles relate to post-traumatic stress disorder (PTSD).

This study focused on a Chinese pediatric population and employed culturally adapted versions of the Revised PTG inventory for children (22) and Coping with Disease scale (23), both of which have been validated for use with Chinese children with chronic illnesses (7, 14). The study aims to examine the coping strategies adopted by children and adolescents newly diagnosed with leukemia across different treatment phases, the dynamic trajectories of their post-traumatic growth, and the interrelationships between these two constructs. By analyzing the dynamic changes in coping styles and PTG levels and their correlations throughout the treatment processes, this study aims to provide empirical evidence and theoretical guidance for developing psychosocial interventions for promoting coping strategies tailored to children with leukemia and enhancing post-traumatic growth in this patient population.

## 2 Materials and methods

### 2.1 Participants and procedures

Sixty children with leukemia who received treatment in the Hematology and Oncology Department of a tertiary hospital in Sichuan Province between May 2022 and August 2023 were selected as research participants. Inclusion criteria for the study participants were as follows: (1) newly diagnosed patients confirmed with leukemia according to the gold standard (24); (2) age between 8 and 18 years; (3) basic reading ability; (4) informed consent obtained from both the patient and their guardian. Exclusion criteria for the study participants: (1) patients unaware of their condition; (2) patients with impaired consciousness or severe mental illness; (3) patients with other serious diseases or treatments, such as malignant bone tumors or solid tumors; (4) critically ill patients.

Data were collected at three time points: T1, before the initial diagnosis and induction of remission; T2, after the induction of remission but before consolidation therapy; and T3, after consolidation therapy but before maintenance treatment. Before the survey, the researchers explained the purpose to the patients and their guardians, obtained their consent, and conducted the survey when the patients were conscious and their conditions were relatively stable. Standardized instructions were used to explain the requirements for completing the questionnaire, and one-on-one guidance was provided to ensure that patients completed the questionnaire accurately. The questionnaires were collected immediately after completion.

Based on the sample size calculation formula for a single-group repeated sampling trial design (25) and with reference to the results of Zhang et al. (26), taking into account the potential loss to follow-up of 10–20%, the required sample size was 60 patients. Overall, 60 patients were included in this study, and the effective sample sizes were 60, 60, and 55 at T1, T2, and T3, respectively. At T3, five patients died, resulting in a dropout rate of 8.33% and an effective recovery rate of 91.67%.

### 2.2 Measures

#### 2.2.1 General information questionnaire

This study was designed by our research team and included sociodemographic data (sex, age, type of residence, family type, parenting style, and parental education level) and disease-related data (disease risk, treatment method, and adverse reactions to disease treatment).

#### 2.2.2 Revised PTG inventory for children

The Revised Post-traumatic Growth Inventory for Children, developed by Kilmer et al. (27) based on the adult PTG inventory. The scale consists of 10 items covering five dimensions: Relating to Others, New Possibilities, Personal Strength, Spiritual Change, and Appreciation of Life. Each item is scored from 0 to 4, with the total score ranging from 0 to 30. Higher scores indicated higher levels of PTG. This scale has been applied across multiple countries in post-traumatic contexts and demonstrated good internal reliability (α = 0.85) and temporal stability (r = 0.44) (28, 29). The Chinese version of the scale was translated and culturally adapted by Yu et al. in Hong Kong (22), who used it to measure post-traumatic growth in children after earthquakes, reporting a Cronbach’s α of 0.86. The scale has also been validated by Liu et al. (7, 14) for use among Chinese adolescents with chronic diseases. In the current study, the Cronbach’s α for this scale was 0.892.

#### 2.2.3 Coping with disease

The Coping with Disease scale, developed by Petersen et al. (30), is designed for children aged 8–18 with chronic diseases. The scale consists of 28 items categorized into six subscales: Acceptance, Cognitive–Palliative, Wishful Thinking, Distance, Emotional Reaction, and Avoidance. Among these, “Acceptance”, “Wishful Thinking”, and “Cognitive–Palliative” are categorized as positive coping styles, while “Emotional Reaction” and “Avoidance” are negative coping styles, and “Distance” is in a neutral state (31). The scale uses a Likert 5-point rating method, with higher scores indicating a greater preference for the corresponding coping style. The Chinese version of the Coping with Disease scale, translated and revised by Li et al. (23) in 2008, was adopted in this study. The original instrument reported an overall Cronbach’s α of 0.83, with the values for the six subscales ranging from 0.64 to 0.88 (23). In the current study, the full scale demonstrated a Cronbach’s α of 0.87, while the subscales yielded α values between 0.74 and 0.88.

### 2.3 Statistical analysis

Data analysis was performed using IBM SPSS Statistics for Windows, version 26.0 (IBM Corp., Armonk, N.Y., USA). Quantitative data were expressed as mean ± standard deviation ( $\bar{x}$ ± s) while categorical data were presented as frequencies and percentages. Missing data from patients lost to follow-up during T3 were imputed using the last-observation carry-over method. Analysis of variance was used to compare the total and subscale scores of the Post-traumatic Growth Inventory for Children at the three time points and the subscale scores of the Coping with Disease. The correlation between the two sets of data was analyzed using Pearson correlation, with a significance level of α = 0.05.

### 2.4 Research ethics

This study was reviewed and approved by the hospital ethics committee (approval number: 2022 [222]). During the investigation, the wishes of the children and their guardians were respected to ensure that informed consent was obtained. Data collection was conducted during the stable phase of the child’s illness, and the duration of data collection was controlled to avoid fatigue or exhaustion. If a child’s condition changed, data collection was immediately halted, and relevant medical interventions were taken by healthcare professionals, adjusting the timing of subsequent data collection accordingly. All collected data were strictly confidential and used solely for scientific research.

## 3 Results

### 3.1 General information about the research participants

This study included 60 patients. All children received chemotherapy at T1 and T2; 85.5% received chemotherapy at T3, and 14.5% underwent hematopoietic stem cell transplantation. In the early stages of treatment, none of the children experienced adverse reactions. However, as they received chemotherapy or hematopoietic stem cell transplantation, adverse reactions began to occur. Other general data are presented in **Table 1**.

**Table 1:** General information on the survey participants (n = 60).

Variable	Category	n	%
Age (years)	8∼12	37	61.7
	13∼18	23	38.3
Gender	Male	33	55.0
	Female	27	45.0
Residence	Urban	20	33.3
	County/Town	15	25.0
	Rural	25	41.7
Family Type	Single-parent	10	16.7
	Nuclear family	17	28.3
	Extended family	33	55.0
Parenting Style	Authoritative	37	61.7
	Authoritarian	15	25.0
	Other	8	13.3
Father’s Education	Primary or below	15	25.0
	Middle-High school	27	45.0
	College or above	18	30.0
Mother’s Education	Primary or below	15	25.0
	Middle-High school	27	45.0
	College or above	18	30.0
Father’s Occupation	Unemployed	6	10.0
	Laborer	33	55.0
	Office worker/Business owner	21	35.0
Mother’s Occupation	Unemployed	19	31.7
	Laborer	23	38.3
	Office worker/Business owner	18	30.0
Payment Method	Self-pay	20	33.3
	Health insurance	40	66.7
Commercial Insurance	Yes	5	8.3
	No	55	91.7
Monthly Household Income (per capita, CNY)	< 1000	16	26.7
	1000 - 2999	26	43.3
	3000 - 4999	14	23.3
	> 5000	4	6.7
Disease Risk	Low-risk	13	21.7
	Intermediate-risk	33	55.0
	High-risk	14	23.3

### 3.2 PTG and coping style scores of children with leukemia

#### 3.2.1 PTG scores of children with leukemia

**Figure 1:**
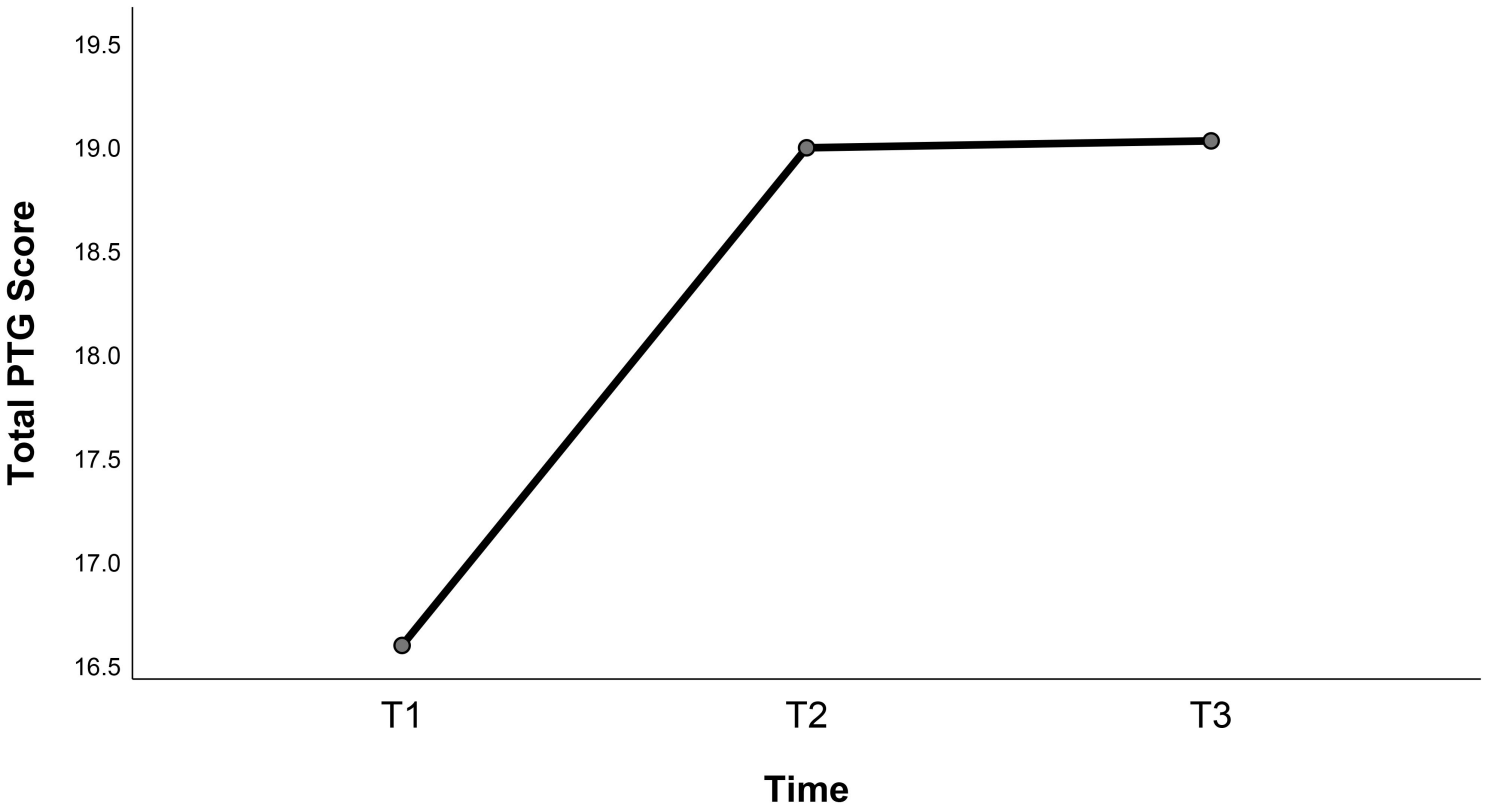
Trajectory trends of total post-traumatic growth scores.

A one-way repeated-measures ANOVA showed significant statistical differences in the total PTG and New Possibility dimension scores of children with leukemia at the three time points (*P* < 0.05) (**Table 2**). Further multiple comparisons using Bonferroni correction indicated that the total PTG score of children with leukemia at T2 was statistically different from that at T1 (*P* < 0.05). Additionally, the new possibility dimension scores at T2 and T3 were significantly different from those at T1 (*P* < 0.05). The trends in changes in the total PTG score trajectory are shown in **Figure 1**.

**Table 2:** Comparison of PTG and coping style scores in children with leukemia (n = 60).

Scale	Subscale/Total	T1	T2	T3	*F-*value	*P*-value
PTG	Total Score	16.58 ± 6.83	18.98 ± 5.90	19.02 ± 6.34	4.320	0.018
	Relating to Others	3.53 ± 1.58	4.15 ± 1.51	3.82 ± 1.48	2.901	0.063
	New Possibilities	2.82 ± 1.75	3.73 ± 1.59	3.72 ± 1.54	6.455	0.003
	Personal Strength	3.30 ± 1.78	3.40 ± 1.56	3.72 ± 1.47	2.932	0.061
	Spiritual Change	3.47 ± 1.61	3.73 ± 1.60	3.83 ± 1.53	1.008	0.371
	Appreciation of Life	3.47 ± 1.79	3.98 ± 1.44	3.93 ± 1.52	2.171	0.146
Coping Style	Acceptance	21.32 ± 5.74	23.12 ± 5.14	23.37 ± 5.37	4.602	0.012
	Wishful Thinking	13.73 ± 1.90	13.85 ± 1.93	13.88 ± 1.94	0.185	0.825
	Cognitive–Palliative	17.35 ± 0.60	18.15 ± 0.56	18.42 ± 0.56	1.299	0.281
	Avoidance	7.85 ± 3.40	7.67 ± 3.47	8.58 ± 3.58	2.076	0.135
	Negative emotional response	11.43 ± 3.74	11.63 ± 4.46	11.45 ± 3.43	0.117	0.890
	Distance	10.57 ± 4.41	11.30 ± 4.56	11.65 ± 4.58	1.984	0.143

#### 3.2.2 The coping style scores of children with leukemia

The results of the one-way repeated-measures ANOVA showed statistically significant differences (*P* < 0.05) in Acceptance dimension scores at different time points among the respondents, as shown in **Table 2**. Further comparison using Bonferroni’s test revealed a statistically significant difference (*P* < 0.05) in the scores of the acceptance dimension at T3 compared to T1.

#### 3.2.3 Correlation analysis between coping style at three time points and PTG in children with leukemia

Across the three treatment phases, the scores for the Acceptance, Cognitive–Palliative, and Distance dimensions of the children’s coping with leukemia scale were all positively correlated with the total PTG score (*P* < 0.05). At T2 and T3, the Avoidance dimension of children with leukemia was positively correlated with the total PTG score and all dimensions except Appreciation of Life (*P* < 0.05). At T3, the Emotional Reaction dimension was negatively correlated with the total PTG score, the Relating to Others dimension, and the Personal Strength dimension (*P* < 0.05), as shown in **Table 3**.

**Table 3:** Correlation analysis between coping style and PTG in children with leukemia (n = 60).

PTG	Acceptance			Wishful Thinking			Cognitive–Palliative		
	T1	T2	T3	T1	T2	T3	T1	T2	T3
Total Score	0.326*	0.473**	0.664**	0.169	0.224	0.254	0.269*	0.498**	0.462**
Relating to Others	0.130	0.446**	0.505**	0.175	0.143	0.418**	0.220	0.418**	0.449**
New Possibilities	0.213	0.167	0.455**	-0.025	0.106	0.123	0.121	0.257*	0.289*
Personal Strength	0.495**	0.392**	0.643**	0.236	0.264*	0.395**	0.316*	0.478**	0.560**
Spiritual Change	0.392*	0.375**	0.636**	0.073	0.236	0.135	0.311*	0.404**	0.431**
Appreciation of Life	0.297*	0.297*	0.430**	0.239	0.111	0.083	0.174	0.368**	0.272*
PTG	Distance			Avoidance			Negative emotional response		
	T1	T2	T3	T1	T2	T3	T1	T2	T3
Total Score	0.298*	0.603**	0.486**	0.162	0.383**	0.348**	-0.073	-0.127	-0.259*
Relating to Others	0.201	0.460**	0.320*	0.079	0.348**	0.400**	-0.065	-0.091	-0.281*
New Possibilities	0.137	0.441**	0.388**	0.133	0.275*	0.193	-0.064	0.021	-0.201
Personal Strength	0.372**	0.430**	0.380**	0.154	0.337**	0.344**	-0.105	-0.139	-0.370**
Spiritual Change	0.245	0.496**	0.507**	0.254*	0.313*	0.380**	-0.064	-0.061	-0.168
Appreciation of Life	0.218	0.364**	0.315*	0.091	0.240	0.109	-0.049	-0.108	-0.146

*P <0.05, **P <0.01.

## 4 Discussion

### 4.1 General data analysis of children with leukemia

Sixty children with leukemia were enrolled in this study. Although previous research has identified 1–4 years as the peak age range for leukemia onset (32, 33), our findings revealed a higher proportion (61.7%) of patients were diagnosed between 8 and 12 years of age, highlighting the need for increased attention to school-aged children with leukemia. The male-to-female ratio in the cohort was 1.22:1, consistent with the demographic patterns reported by Liu et al. (33) in a retrospective analysis of pediatric leukemia cases in Sichuan and Chongqing, China. Notably, a majority of participants resided in rural areas, where primary caregivers exhibited lower educational attainment, unstable employment, and reduced income levels. Over half of the families relied on health insurance to cover their medical expenses. These socioeconomic barriers align with the findings of Wu et al. (34), who demonstrated that younger age, parental illiteracy, and financial strain collectively impair caregiving capacity. To mitigate these challenges, healthcare providers should prioritize targeted interventions, including disease education and skill-building programs for caregivers to enhance treatment adherence and improve clinical outcomes.

### 4.2 Dynamic changes of PTG scores in children with leukemia

The results of this study showed that the PTG of the children during the treatment period was at a medium level, using the mean score of the scale as a cut-off point. This may be owing to the young age of the study participants; their cognitive development is not mature enough for them to independently manage their illness or fully understand the positive meaning of the negative event of illness in their lives. Another contributing factor may be the stress and trauma experienced by parents following the diagnosis, which can limit their ability to take care of them. As a result, the children may receive insufficient and lower-quality social support, negatively affecting their psychological adjustment and hindering PTG. The results of this study are consistent with the findings of Qian et al. (7), who investigated the PTG of children and adolescent cancer survivors, suggesting that although children with leukemia have gained positive results in their fight against the disease, PTG still has a great deal of room for development. PTG shows dynamic changes with traumatic events, time, and other factors (12). The total PTG scores of the survey respondents from T1 to T2 showed an increasing trend, which may be attributed to the fact that children and their families are often in a state of stress at the early stage of disease diagnosis (35), and awareness of the disease and coping strategies have not yet been formed, resulting in lower PTG scores. With the advancement of treatment, children and parents gradually adapt to the changes brought about by the disease and actively seek coping strategies, and post-traumatic psychology gradually produces positive growth.

The rate of PTG development slowed down from T2 to T3, which may be attributed to the heavier economic and psychological burdens on the caregiver. These challenges can strain good parent-child relationships and reduce attention to children’s psychological needs, thereby hindering their PTG (36). Further studies have shown that adults with cancer receive positive thinking therapy (37) and psychotherapy (38) to enhance PTG. However, there are few intervention studies on PTG in children with leukemia, which may be the reason for the lower long-term PTG scores observed in the population. In addition, factors related to individuals’ awareness of vulnerability can hinder PTG (39). With prolonged disease duration, children with leukemia experience a variety of symptoms related to chemotherapy and may be worried about their future, which can affect their mental health. In this study, the scores on the personal strength and spiritual change dimensions of children with leukemia at the three time points showed a continuous upward trend, while the scores on relating to others, new possibilities, and appreciation of life dimensions showed an inverted U-shaped trend, initially increasing and then decreasing. This may be because personal strength and spiritual change rely more on introspective processes. After the age of eight, children have increasing cultural knowledge, a preliminary understanding of society and personal thoughts, and a gradual increase in personal autonomy, cognitive level, and judgmental ability. The other three dimensions are more related to external interactions and are susceptible to parental care stress, parent-child relationships, disease regression, and impaired self-image (40). Medical personnel are encouraged to utilize evidence-based psychosocial care standards for childhood cancer and develop tailored intervention plans aligned with current clinical realities (41). From a family systems perspective, empirically supported psychosocial interventions (42) such as cognitive behavioral therapy and family systems therapy are encouraged to mitigate caregivers’ post-traumatic stress symptoms, actively engage family members in the treatment process, and foster constructive parent–child relationships (36). Based on the theoretical basis of positive psychology ideas, they can formulate a positive psychological group counseling program in the same ward (43), which can guide children’s self-healing, improve their negative emotions and psychological state, and enhance their PTG.

### 4.3 Types of coping style in children with leukemia

A positive coping style is a crucial factor in an individual’s successful adaptation and a prerequisite for PTG (20, 44). Acceptance, Cognitive–Palliative, and Wishful Thinking are considered positive coping styles, whereas Avoidance and Emotional Reactions are categorized as negative coping styles (31). In this study, the most commonly used coping style among patients was “Acceptance,” followed by “Cognitive–Palliative,” with “Avoidance” having the lowest frequency of use. Additionally, scores on the dimensions of Wishful Thinking, Acceptance, and Cognitive–Palliative increased gradually at the three time points, which may be owing to the diagnosis of illness and changes in living conditions, prompting patients to reflect on their health and life changes, thereby alleviating psychological stress after trauma, altering maladaptive thought patterns related to trauma, and reducing negative emotions. This may also be related to the social support received from family, friends, and other sources, leading patients to adopt more positive coping styles. Farsi (45) pointed out that the coping process is non-linear and that multiple factors, such as levels of anxiety and depression, situational factors, and coping skills, influence patients’ coping styles. In this study, the score on the Avoidance dimension decreased from high to low and then increased, whereas the score on the Emotional Reaction dimension decreased from low to high and then decreased. This may be owing to fluctuations in avoidance and negative emotions, possibly related to the younger age of the patients in this study, along with their weaker emotional processing abilities and interpersonal skills. As the disease progresses, the intensity of the stimuli experienced by patients may change, leading them to adopt a more negative coping style. In addition, a study by Lin et al. (46) found that adult patients with acute leukemia primarily adopted two coping styles: Avoidance and Submission, which is inconsistent with the results of this study. A possible reason for this is that adult patients with an initial diagnosis of leukemia differ in age and way of thinking from children, leading to different coping methods. Therefore, healthcare professionals should develop comprehensive and patient-centered coping strategies tailored to children with leukemia, actively encourage parental involvement, and promote the adoption of positive coping mechanisms among these patients. Yang et al. showed that the coping style of children with chronic diseases is influenced by multiple factors, including the child’s age, condition, whether they have taken a leave of absence, and their place of residence (31). This suggests that medical personnel should pay attention to the coping behaviors of children with leukemia, particularly those who experience recurrent conditions, prolonged hospital stays, take a leave of absence, or live in rural areas. They should promptly guide and alleviate the negative emotional responses of these children, encourage them to adopt positive coping styles such as facing, accepting, and self-comforting.

### 4.4 Correlation analysis between coping style and PTG scores

This study showed that the scores on the Acceptance, Cognitive–Palliative, and Distance dimensions in the coping style scale at the three time points were positively correlated with the total PTG score. These results are largely consistent with existing research findings (7, 14), indicating that the more actively children adopt a coping style in their struggle against illness, the more likely they are to experience positive psychological growth after trauma. This suggests that healthcare providers should develop intervention measures to guide children to adopt proactive coping styles, such as psychological counseling, cognitive-behavioral therapy, or mindfulness training (37, 38), to help children accept changes brought about by illness, and to guide them to regulate their emotions through self-soothing (e.g., positive self-talk).

After inducing remission and before maintenance therapy, the Avoidance dimension of patients with leukemia was positively correlated with the total PTG score and with each dimension (except for appreciation of life). This finding is consistent with the conclusions of Lu et al. (14). However, it differs from the findings of Prati et al. (47), indicating that the mechanism by which avoidance coping affects PTG remains controversial and warrants further research. Active coping is not entirely independent of passive coping; the effects of passive coping may also manifest through interactions with active coping (45). This finding suggests that healthcare providers should pay attention to the diversity and interactivity of coping styles when guiding patients with leukemia. They should actively intervene in patients who use passive coping and appropriately encourage those who use active coping to promote positive psychological development.

After consolidation therapy and before maintenance treatment, the negative emotional response dimensions were negatively correlated with the total PTG score, interpersonal relationship dimension, and personal strength dimension. This is consistent with the findings of Peles (48), indicating that children with leukemia tend to adopt a passive coping style as their condition progresses and treatment advances, potentially hindering PTG. Possible reasons for this result include the prolonged treatment faced by newly diagnosed patients, particularly chemotherapy, and the significant discomfort caused by the disease itself. Coupled with concerns about prognosis and adverse outcomes, these factors can easily lead to feelings of helplessness and frustration, affecting their confidence and courage to overcome the illness, thereby increasing psychological vulnerability. This suggests that in clinical practice, medical staff should pay attention to the psychological state of patients, be vigilant against adopting negative emotions or other passive methods, and use various approaches, such as pain management (49) and art therapy (50), to guide and encourage them, promoting positive coping and a more optimistic outlook. Furthermore, eye movement desensitization and reprocessing (EMDR) therapy serves as an effective and innovative therapeutic approach (51, 52). Healthcare professionals can draw on international expertise to develop localized standard protocols addressing past, present, and future aspects, thereby guiding pediatric patients in coping with the physical challenges and unpredictable nature of the disease.

This study shows that the PTG levels of children and adolescents with newly diagnosed leukemia vary dynamically at different stages of disease treatment; “Acceptance” was the most frequently used coping strategy, while “Avoidance” was relatively the least used. Using a positive coping style (Acceptance, Cognitive–Palliative, and Distance) helped patients achieve positive psychological changes after trauma, whereas a negative coping style (Emotional Reactions) hinders post-traumatic psychological development. Therefore, healthcare providers should comprehensively and scientifically assess the coping styles and behaviors of patients at various points in time, particularly those with longer disease duration and extended hospital stay. Effective coping styles should be developed to alleviate patients’ negative emotions, encourage family involvement, and provide adequate psychosocial interventions to promote PTG. This study has some limitations. First, the sample size was small and limited to one hospital. Future multi-center collaborative studies with larger, more diverse samples are needed; this will not only enhance statistical power but also allow for the investigation of important moderators and ensure better representation of vulnerable or underserved subpopulations within the pediatric oncology community. Second, the measurement time points were based on the treatment plans of newly diagnosed patients. Future research could choose different measurement time points based on relevant theoretical foundations to further understand the characteristics of coping style and the trends in PTG, thereby enriching the study of the mechanisms of PTG. Third, future research could use latent profile analysis to characterize post-traumatic growth among children with leukemia across different subtypes or age groups, with a focus on interindividual differences.

## Data Availability

The original contributions presented in the study are included in the article/supplementary material. Further inquiries can be directed to the corresponding author.
